# Case of Pedunculated Fibroid Removed

**Published:** 1901-01

**Authors:** C. Carr


### Clinical Report.

### CASE OF PEDUNCULATED FIBROID REMOVED.

By L. G. HANLEY, M. D., Buffalo, N. Y.,
Gynecologist Hospital of the Sisters of Charity.

Reported by C. CARR, M. D.

MISS R., aged 33; single; admitted to hospital November 3, 1900, suffering from pain attributed to a large swelling in the abdomen. Two weeks prior to admission, had been under care of a physician, who recommended operation.

Examination revealed a solid tumor in the pelvis, extending three inches above the umbilicus and lying to the right of the median line. Temperature 101½°; tumor immovable and extremely tender. Her bowels were constipated and she suffered occasional attacks of vomiting. There was an area seven inches in diameter that was very painful to touch over upper part of tumor.

Patient scientifically prepared and operation performed under rigid surgical cleanliness. On opening the abdomen the peritoneum, omentum and serosa of tumor were found to adhere firmly in a solid mass. Twelve inches of the ileum were agglutinated to tumor. The omentum was separated, which was found to be gangrenous in parts. Peritoneum showed the effects of severe inflammatory action. The omentum was separated and diseased parts removed.

The tumor was drawn up into abdominal opening and the bowel dissected from it, not, however, without leaving the serous covering where the bowel was attached adherent to bowel, thus avoiding any rupture or injury to the gut.

The specimen being freed, it was easy to see the cause of trouble, which proved to be a solid tumor with pedicle growing from the right cornu of the uterus. The pedicle, only one-quarter of an inch in diameter, was twisted and in a gangrenous condition. This was clamped and tumor removed.

It was necessary to make a wedge-shaped incision in the uterus, which was closed with catgut. The ovary on the left side was in a state of cystic degeneration, about the size of a small orange. This was removed. The abdominal cavity was filled with a normal saline solution and the wound closed. Peritoneum closed with catgut and the remaining tissues en masse with silkworm-gut.

Weight of tumor, eight pounds. The day following operation, morning temperature 99°, vomiting ceased. The sutures were removed on the thirteenth day, when union by adhesion had taken place. The patient made an uninterrupted recovery. Sat up on the twentieth day.

